# Pancreatic ductal adenocarcinoma in colonic wall: metastatic disease or cancerized pancreatic ectopic tissue?

**DOI:** 10.1186/s40792-020-00846-5

**Published:** 2020-04-22

**Authors:** Graziana Gallo, Alessandro Mangogna, Gianrocco Manco, Stefania Caramaschi, Tiziana Salviato

**Affiliations:** 1 Department of Diagnostic, Clinic and Public Health Medicine, University of Modena and Reggio Emilia, Modena, Italy; 2 Department of Medical, Surgical and Health Science, University of Trieste, Cattinara Hospital, Strada di Fiume, 447, 34149 Trieste, Italy; 3 Department of Surgery, University of Modena, Modena, Italy

**Keywords:** Ectopic pancreas, Colon, Adenocarcinoma

## Abstract

We describe two unusual cases of cancerized ectopic pancreatic parenchyma within the wall of the left colon. Although the morphology of the neoplastic cells and their immunoprofile were consistent with pancreatic ductal adenocarcinoma, the detection of small foci of regular ectopic pancreatic tissue close to dysplastic glands at the periphery of the cancerized mass represented the key diagnostic features. A careful histological examination of surgical samples represents the correct approach to the diagnosis of this rare disease, mostly when total-body CT scan evaluation confirms the lack of bilio-pancreatic masses.

To the Editor,

We read with interest the paper by Kaneko et al. [1] reporting an unusual case of malignant transformation of an ectopic pancreas tissue in the duodenum, presenting with vomiting caused by obstruction. In that particular case, the tumor showed no anatomic connection with the normal pancreas, yet the detection of normal pancreatic tissue adjacent to the tumor mass along to its immunohistochemical profile suggested the diagnosis of ductal adenocarcinoma arising from ectopic pancreas [1, 2].

The Kaneko et al. article prompted us to report two cases of pancreatic ductal adenocarcinoma which occurred in the colonic splenic flexure of a 74-year-old male (Fig. 1a–c) and in the sigmoid colon of a 78-year-old female, respectively (Fig. 1e–g). Clinically, both patients presented with abdominal pain, positive for fecal occult blood test, and general malaise. Colonoscopies showed irregular mucosal thickness respectively of 3.5 and 7 cm in length and erythematous surface. Due to suspected colonic malignancy, both patients underwent biopsy that revealed no evidence of malignancy. Subsequent surgical resection was performed. Gross examination revealed irregular white surfaces. Microscopic evaluation of routinely stained tissue sections features disorderly, sometimes hyperplastic glands with hyperchromatic cells within a desmoplastic stroma. Atypical glands were immunoreactive for CDX2, CK7, and monoclonal CEA but negative for CK20. The overall findings were deemed consistent with ductal adenocarcinoma and suggested cancerized ectopic pancreatic tissue in colonic wall (Fig. 1 d and h). Total-body computed tomography (CT) scan excluded other malignancies. Biomolecular analysis of our cases detected *KRAS* mutation. After a follow up of 3 years, both patients are fine, without recurrence.

**Fig. 1:**
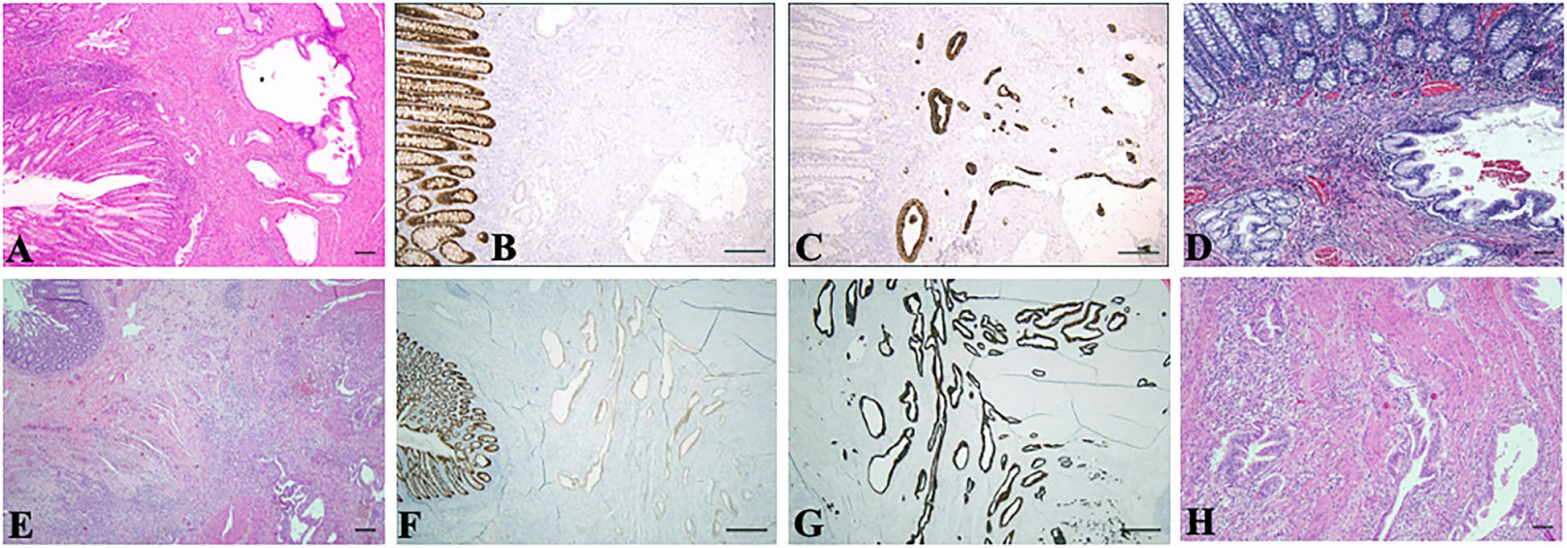
Case of a 74-year-old male. **a** Well differentiated pancreatic ductal adenocarcinoma near dilated, pancreatic gland in the bowel wall exhibiting architectural and cytologic atypia [hematoxylin and eosin (H&E) stain; 4×]. **b** Tumor cells are negative for CDX2 in neoplastic glands, compared to normally immunoreactive colonic mucosa (10×). **c** Complementary CK7 positivity of neoplastic glands and negativity of the non-neoplastic colonic mucosa (10×). **d** Atypical pancreatic glands close to normal pancreatic glands (H&E stain; 4×). Case of a 78-year-old female. **e** Well differentiated pancreatic ductal adenocarcinoma near to the pancreatic gland in the bowel wall (H&E stain; 4×). **f** Immunohistochemistry (IHC) shows negativity for CDX2 in neoplastic glands associated to positivity in the normal colonic mucosa (10×). **g** IHC shows positivity for CK7 in neoplastic glands associated to negativity in the normal colonic mucosa (10×). **h** Dysplastic pancreatic glands (H&E stain; 4×). Scale bars 100 μm

By definition, ectopic pancreas is defined as pancreatic tissues having no anatomic or vascular connection with the orthotopic pancreas [3, 4]. The diagnosis of ectopic pancreas cancerization is difficult: in fact, the identification of a normal pancreatic component (either glands or acini) within or near the tumor area may not always be possible [2, 5]. Furthermore, clinical features and case imaging lack specificity. Pathologic examination of tissue specimens remains therefore of critical importance.

Among few cases reported in literature, the most frequent occurrences of ductal adenocarcinoma from ectopic pancreatic tissue included mainly the stomach [5]. It is rare in the duodenum and jejunum: only 23 cases have been described in literature [1, 5, 6]. It is even rarer in the mesocolon or rectum wall [5–7]. In all cases, the diagnosis was rendered based on combined CT scan images and microscopic features.

In conclusion, cancerization of ectopic pancreatic parenchyma is a rare event as long as other primary tumors arising within the pancreas, gastro-intestinal, or biliary tract are excluded. However, our cases in the colon make us think that this pathology can be underestimated.

## Data Availability

The dataset supporting the conclusions of this letter is included within the letter.

## Abbreviations

CDX2: Caudal type homeobox 2
CEA: Carcinoembryonic antigen
CK: Cytokeratin
CT: Computed tomography
KRAS: Ki-ras2 Kirsten rat sarcoma viral oncogene homolog

## References

[1] Kaneko T, Ohara M, Okamura K, Fujiwara-Kuroda A, Miyasaka D, Yamabuki T (2019). Adenocarcinoma arising from an ectopic pancreas in the duodenum: a case report. Surg Case Rep..

[2] Guillou L, Nordback P, Gerber C, Schneider RP (1994). Ductal adenocarcinoma arising in a heterotopic pancreas situated in a hiatal hernia. Arch Pathol Lab Med..

[3] Jaervi O, Lauren P (1964). Gastric glandular tumors provided with excretory ducts, and criticism of the theory of the tumors arising in heterotopic pancreas; observations on the occurrence of atypical glands in the stomach. Acta Pathol Microbiol Scand..

[4] Thoeni RF, Gedgaudas RK (1980). Ectopic pancreas: usual and unusual features. Gastrointest Radiol..

[5] Cazacu IM, Luzuriaga Chavez AA, Nogueras Gonzalez GM, Saftoiu A, Bhutani MS (2019). Malignant transformation of ectopic pancreas. Dig Dis Sci..

[6] Zhang P, Wang M, Bai L, Zhuang W (2019). A unique case of ectopic pancreas presenting as jejunal malignance. J Surg Case Rep.

[7] Goodarzi M, Rashid A, Maru D (2010). Invasive ductal adenocarcinoma arising from pancreatic heterotopia in rectum: case report and review of literature. Hum Pathol..

